# Presence of atrial fibrillation is associated with liver stiffness in an elderly Finnish population

**DOI:** 10.1371/journal.pone.0173855

**Published:** 2017-03-13

**Authors:** Aki Juhani Käräjämäki, Olli Kettunen, Samuli Lepojärvi, Olli-Pekka Koivurova, Y. Antero Kesäniemi, Heikki Huikuri, Olavi Ukkola

**Affiliations:** 1 Research Unit of Internal Medicine, Medical Research Center Oulu, Oulu University Hospital, and University of Oulu, Oulu, Finland; 2 Abdominal Center, Department of Internal Medicine, Oulu University Hospital, Oulu, Finland; Texas A&M University, UNITED STATES

## Abstract

**Background:**

Chronic liver injury from different etiologies drives liver fibrosis. However, little is known about the associated factors, systemic factors in particular. Recently, non-alcoholic fatty liver disease (NAFLD) and atrial fibrillation have been shown to be associated with each other. Thereby, we aimed to study the association between atrial fibrillation and liver stiffness.

**Study:**

Extensive clinical measurements including echocardiography of the heart, transient elastography (TE) of the liver and the presence of atrial fibrillation were determined in elderly Finnish study subjects (n = 76, mean age 73 years) from OPERA (Oulu Project Elucidating the Risk of Atherosclerosis) study cohort. Half of the study subjects had non-alcoholic fatty liver disease, whereas others did not have any known hepatic morbidity. The present study was cross-sectional by nature.

**Results:**

The subjects with atrial fibrillation had higher TE values (with atrial fibrillation TE = 9.3kPa, without atrial fibrillation TE = 6.3kPa, p = 0.018). When the cohort was divided to four subgroups (those without NAFLD or atrial fibrillation, with NAFLD but without atrial fibrillation, with both conditions, and with atrial fibrillation but without NAFLD), the TE value was the highest in the subjects with both conditions (5.3kPa, 7.4kPa, 10.8kPa and 7.8kPa, respectively, p = 0.019). Moreover, the higher the TE value, the more prevalent atrial fibrillation was (the atrial fibrillation prevalence by tertiles of TE 27% / 36% / 77%, p = 0.001). Likewise, the greater the TE value, the greater the left atrial diameter, a collateral of atrial fibrillation (left atrial diameters by tertiles of TE 39mm / 45mm / 48mm, p<0.001) was. All these p-values for continuous variables remained statistically significant even after adjustment for common clinically relevant risk factors.

**Conclusions:**

There is an association between atrial fibrillation and liver stiffness. This novel association may have multiple explanations and mechanistic links, which are discussed here and need further studies, prospective studies in particular.

## Introduction

Chronic liver injury from different etiologies causes chronic liver inflammation and, thus, drives the formation of liver fibrosis [1, 2]. Liver fibrosis is considered as the first common step of different liver diseases toward cirrhosis and its complications, such as liver failure, hepatocellular carcinoma and death [2, 3]. It is preventable and at least partially reversible if the underlying etiology is removed [1–3]. For instance, in the general Dutch population the prevalence of clinically relevant liver fibrosis is 5.6% and it is strongly associated with higher age, steatosis and diabetes [4]. In order to develop new therapeutic innovations, investigation of the mechanisms driving liver fibrosis is of the utmost importance.

Atrial fibrillation (AF) is the most prevalent cardiac arrhythmia and, due to ageing of the population, its prevalence is expected to rise in the future [5, 6]. The association between non-alcoholic fatty liver disease (NAFLD) and AF has recently been presented [7, 8]. The mechanisms that link these two entities are not completely understood, but it is reasonable to assume that this association is causal [7] and two-way. Thus, we investigated whether the presence of AF, and its collateral, expanded left atrium diameter (LAD), correlate with liver stiffness, estimated by transient elastography (TE) levels [9], in an elderly Finnish population.

## Materials and methods

### Study cohort

The OPERA study (Oulu Project Elucidating the Risk of Atherosclerosis) was initiated in the early 1990s. It focused on the research of atherosclerosis and its co-morbidites.

All OPERA study subjects visited the research laboratory of the Department of Internal Medicine, University of Oulu, where they underwent a comprehensive clinical examination with, for instance, ultrasonography examination of the upper stomach by one experienced radiologist. He evaluated the presence of hepatosteatosis with the help of the liver-kidney contrast [10]. The first phase of the study was conducted between the years 1991 and 1993. The baseline examinations are reported in detail elsewhere [7, 11].

In the second phase of the study, all participants were invited to attend a follow-up visit, converting OPERA into a prospective study. Of the 813 survivors, a total of 600 subjects attended (281 men (47%), mean age 72 years, range 62–83 years, NAFLD in 129 subjects). From these 600 subjects TE was measured for 86 subjects. After excluding those with excess alcohol intake at the baseline or at the follow-up visit and with unreliable TE measurement, 76 subjects were left forming the cohort in the present study. The second phase took place in 2013 and 2014.

### Characteristics of the study cohort

In the present study, the time of the cross-section of our cohort is 2013–2014 (the control visit of the OPERA study). All 76 study subjects visited the research laboratory of the Department of Internal Medicine, University of Oulu, where they underwent a comprehensive clinical examination with anthropometric and blood pressure measurements. There was at least 10 minutes of rest before blood pressure measurements, and three measurements were taken at 1-minute intervals. The means of the last two were used in the analyses. The reported heart rate is from the third measurement. In addition, a wide range of laboratory samples were collected after an overnight fast and a standardized health questionnaire was completed covering the subjects’ medical history, alcohol habits, smoking, physical activity, current and former medication and family history. All these examinations were completed by a specifically trained nurse under the supervision of a physician.

Cardiac echoparameter measurements were taken using a GE Healthcare Vivid E 9 VERSION 110. x.x ultrasound system [12] by one experienced cardiologist who was blinded to OPERA-related data.

The diagnosis of AF (atrial flutter included) was made if ICD-10 code I48 was listed in the patients’ medical records at the follow-up visit. The validity of this method has been shown adequate [13, 14]. The diagnosis of coronary artery disease (CAD) was made if at least one code from the ICD-10 code I20-I25 was listed in the patient’s medical records or if the subject had undergone coronary artery bypass grafting (CABG) or coronary angioplasty (only confirmed CAD diagnosis was accepted, as opposed to suspected CAD diagnosis based only on symptoms). Hypertension was diagnosed if the subject had a previous diagnosis of hypertension or if the blood pressure was ≥ 140/90mmHg (means of the second and third measurements) at the clinical examination. Moreover, we calculated the Quicki for insulin resistance surrogate: Quicki = 1/[log (fasting insulin)+log (fasting glucose)]. The renal function was estimated with CKD-Epi equation [15]. BMI was calculated as weight (kg) divided by height squared (m^2^).

Throughout this study, the subject was thought to have NAFLD if the subject had ultrasonography-diagnosed hepatosteatosis at the baseline of the OPERA study and there was not excess alcohol intake (≥ 210g in a week in men or ≥ 140g in a week in women [16]) at the baseline of the OPERA study or at the time of the follow-up visit. These definition criteria have been largely accepted in the Finnish NAFLD studies [17]. Apart from NAFLD, there were not subjects with any other known hepatic morbidity.

We selected the study subjects for the TE examination [9, 18, 19] evenly from four subgroups: those without NAFLD or AF, with NAFLD but without AF, with both conditions and with AF but without NAFLD. The distribution of the basic characteristics (BMI, age, gender) was aimed to be as similar as possible. The goal was to measure TE from about one hundred subjects, but, after matching the characteristics from the subjects available, there were 86 subjects in total. The examination was performed in 2015 by an experienced gastroenterologist who was not aware of the previous OPERA data. All measurements were done in the morning after an overnight fast. There were at least 10 successful measurements with the M probe, all of them were performed from the right-sided central axis line. Exclusion criteria were excess alcohol intake (≥ 210g a week in men or ≥ 140g a week in women) or other liver disease than NAFLD. After excluding 5 subjects due to excess alcohol intake and 5 subjects due to unreliable TE results (unreliability due to obesity), there were 76 subjects available in the present study.

In the present study, NAFLD Fibrosis Score (NFS) [20] was used to confirm the findings of TE. NFS is validated scoring system consisting of widely used laboratory and clinical data (age, body mass index (BMI), impaired glucose tolerance (IGF) / diabetes, aspartate aminotransferase (AST), alanine aminotransferase (ALT), platelets and albumin). In clinical practice it is used to separate NAFLD patients with and without advanced fibrosis (≥ F3) [20, 21]. NFS was measured of all 129 NAFLD subjects available at the follow-up visit to see whether the association is present in a bigger study cohort and in another liver fibrosis proxy measurement. NFS of the follow-up visit data was calculated with the help of a calculator available on the Internet (www.nafldscore.com).

### Statistics

Statistical analysis was performed using IBM Corp. Released 2013. IBM SPSS Statistics for Windows, Version 23.0. Armonk, NY: IBM Corp. The statistical significances of the differences in continuous variables between the groups were assessed using the analysis of variance (ANOVA). The normal distribution of TE and LAD was tested visually with histogram and with Kolmogorov-Smirnov test, in which the p-value <0.05 was considered to indicate the skewness distribution. In that case, the results were also tested with Mann-Whittney U test or Kruskal-Wallis non-parametric ANOVA test. The statistical significances of the differences in categorical variables were tested by the Chi-Square test. Analysis of covariance was used to compare the associations with adjustments. A p-value <0.05 was considered to be statistically significant.

### Ethics

This study was approved by the Ethics Committee of the Medical Department of Oulu University (48/2009). The participants gave a written informed consent for the use of their clinical records.

## Results

The characteristics of the study subjects with TE measurement available (n = 76) by the presence or absence of AF are shown in Table 1. It shows, for instance, that LAD was greater in the AF subjects than in the non-AF subjects (49 ± 9mm vs. 40 ± 5mm, p<0.001). Besides that, only GGT (p = 0.011), plasma albumin (p = 0.030) and LDL cholesterol (p = 0.023) differed statistically significantly between those with and without AF.

**Table 1:** The characteristics of the study subjects with transient elastography measurement (n = 76) available by AF status.

	AF (n = 36)	non-AF (n = 40)	p-value
Gender, n (%) (men)	29 (81%)	32 (80%)	0.952
Age (years)	73 (± 5)	72 (± 5)	0.285
BMI (kg/m2)	30.2 (± 4)	30.1 (± 4)	0.964
Waist (cm)	103 (± 11)	103 (± 13)	0.920
Systolic blood pressure (mmHg)	132 (± 24)	139 (± 18)	0.128
Diastolic blood pressure (mmHg)	72 (± 11)	74 (± 11)	0.346
Heart rate (/min)	69 (± 14)	70 (± 13)	0.581
Alcohol/week (g)	37 (± 54)	37 (± 48)	0.964
Smoking (pack years)	15 (± 24)	12 (± 20)	0.639
Hypertensives, n (%)	31 (86%)	32 (80%)	0.480
CAD, n (%)	15 (42%)	11 (28%)	0.194
LVMI (g/m^2^)**	122 (± 24)	126 (± 27)	0.534
LAD (mm)**	49 (± 9)	40 (± 5)	<0.001
CKD-Epi (ml/min)	78 (± 19)	85 (± 11)	0.062
Leptin (ng/L)	16 (± 12)	17 (± 19)	0.777
Adiponectin (ug/mL)	14 (± 9)	12 (± 6)	0.226
Resistin (ng/mL)*	13 (± 5)	10 (± 3)	0.108
ALT (U/L)	28 (± 19)	30 (± 16)	0.691
GGT (U/L)	53 (± 31)	35 (± 29)	0.011
Alb (g/L)	39 (± 3)	41 (± 3)	0.030
Cholesterol (mmol/L)	4.2 (± 0.9)	4.5 (± 0.9)	0.137
LDL-cholesterol (mmol/L)	2.4 (± 0.7)	2.8 (± 1.0)	0.023
HDL-cholesterol (mmol/L)	1.3 (± 0.5)	1.3 (± 0.3)	0.992
HbA1C% (%)	6.4 (± 1.1)	6.2 (± 0.9)	0.344
hs-CRP (mg/L)	2.5 (± 2.4)	2.0 (± 2.3)	0.407
TSH (mU/L)	2.7 (± 1.7)	2.4 (± 1.2)	0.370
Quicki (L/mmol)	0.50 (± 0.10)	0.50 (± 0.07)	0.914
ACE inhibitor, n (%)	16 (44%)	13 (33%)	0.284
Lipid lowering drugs, n (%)	25 (69%)	20 (50%)	0.085

The continuous variables are expressed as mean values ± standard deviation and the categorical variables as absolute numbers, with percentage in brackets.

The differences were tested by the ANOVA test for the continuous variables and Pearson Chi-Squared test for categorical variables. The abbreviations are as follows: BMI, body mass index; CAD, coronary artery disease; LVMI, left ventricular mass index; LAD, left atrial diameter; CKD-Epi, Glomerular filtration rate by the Chronic Kidney Disease Epidemiology Collaboration (CKD-EPI) equation; ALT, Alanine aminotransferase; GGT, gamma-glutamyl transpeptidase; Alb, albumin; LDL, Low-density lipoprotein; HDL, High-density lipoprotein; HbA1C%, glycosylated hemoglobin A1C; hs-CRP high sensitive C-reactive protein; TSH thyroid stimulating hormone; Quicki (surrogate for insulin resistance, Quicki = 1/[log (fasting insulin)+log (fasting glucose)]); ACE, angiotensin converting enzyme inhibitor. The reported heart rate is the heart rate of the third measurement of the blood pressure. The reported systolic and diastolic blood pressures are the means of the second and third blood pressure measurements during the control visit. Of 36 subjects with AF (mean duration 109 months), 10 had paroxysmal (mean duration 117 months, range 35–226 months) and 26 chronic (mean duration 106 months, range 27–303 months) AF.

* Data available on 24 subjects

** Data available on 75 subjects.

**Fig 1:**
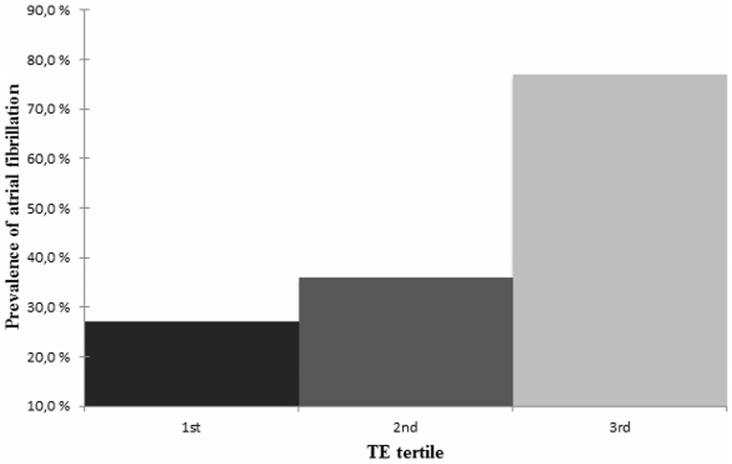
The prevalence of atrial fibrillation by the transient elastography tertiles. There were 6/22 subjects (27%) with atrial fibrillation in the first tertile, 10/28 subjects (36%) in the second tertile and 20/26 subjects (77%) in the third tertile (p = 0.001). Abbreviation: TE, transient elastography.

Fig 1 shows the prevalence of AF by tertiles of TE. According to these figures, the greater the tertile, the greater the AF prevalence (p = 0.001).

**Fig 2:**
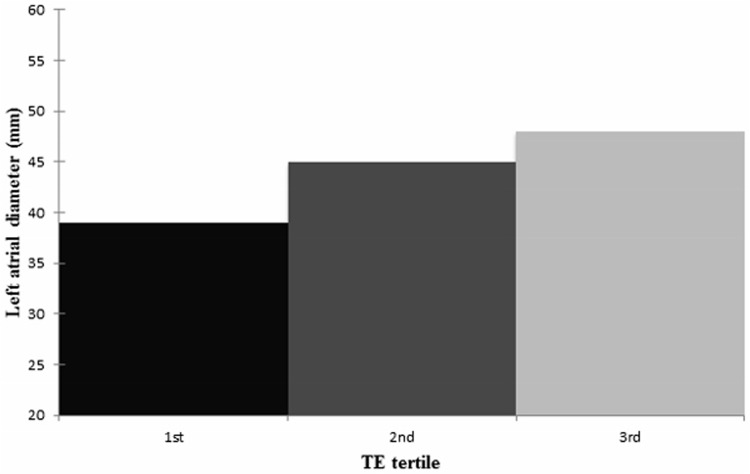
The left atrial diameter (mm) by transient elastography tertiles. The mean LAD in the first TE tertile (n = 21) was 39mm (SD ±7mm), in the second tertile (n = 28) 45mm (SD ± 7mm) and in the third tertile (n = 26) 48mm (SD ± 8mm) (p<0.001). After adjustments (BMI, age, gender, amount of alcohol intake (g/week), smoking (pack years), Quick index, systolic blood pressure) the statistical significance prevailed (p = 0.012). Abbreviations: LAD, left atrial diameter; TE, transient elastography; BMI, body mass index.

The LAD (mm) by tertiles of TE is illustrated in Fig 2. Collaterally to the AF prevalence, LAD tended to increase by the greater tertile before (p<0.001) and after adjustments for the most important co-variates (BMI, age, gender, alcohol intake, smoking, Quicki and systolic blood pressure) (p = 0.012).

The subjects who had experienced AF also had greater TE values compared to those without AF (9.3 kPa vs. 6.3 kPa, p = 0.018). The difference was still evident after adjustments (see above, p = 0.005). Additionally, there were more subjects with clinically relevant fibrosis by TE > 8kPa in subjects with AF (n = 18, 50%) than in subjects without AF (n = 5, 13%) (p<0.001) [4].

The descriptive characteristics of the four subgroups (those without NAFLD or AF, with NAFLD but without AF, with both conditions, and with AF but without NAFLD) are shown in Table 2. It shows that the TE value was the highest in the subjects with both conditions (5.3kPa, 7.4kPa, 10.8kPa and 7.8kPa, respectively) (p = 0.019, after adjustments p = 0.006).

**Table 2:** The characteristics of the subjects with transient elastography (n = 76) by subgroups used in the study selection.

	NAFLD-, AF–(n = 20)	NAFLD+, AF–(n = 20)	NAFLD+, AF+ (n = 18)	NAFLD-, AF+ (n = 18)	p-value
Age	73 (± 6)	71 (± 5)	72 (± 6)	75 (± 5)	0.312
Gender (men). n (%)	16 (80%)	16 (80%)	15 (83%)	14 (78%)	0.981
BMI (kg/m^2^)	30.5 (± 5)	29.8 (± 4)	30.3 (± 5)	30.0 (± 4)	0.967
Alcohol (g/week)	41 (± 52)	34 (± 45)	35 (± 55)	39 (± 54)	0.964
Quicki (L/mmol)	0.50 (± 0.06)	0.50 (± 0.08)	0.50 (± 0.11)	0.50 (± 0.08)	0.995
Systolic blood pressure (mmHg)	138 (± 14)	141 (± 20)	133 (± 28)	131 (± 20)	0.455
Smoking (pack years)	16 (± 25)	9 (± 14)	17 (± 24)	12 (± 24)	0.644
TE (kPa)	5.3 (± 1.8)	7.4 (± 4.8)	10.8 (± 9.0)	7.8 (± 2.5)	0.019
ALT (U/L)	26 (± 10)	33 (± 20)	29 (± 23)	27 (± 14)	0.544
GGT (U/L)	37 (± 36)	34 (± 19)	61 (± 36)	45 (± 23)	0.029

The variables are expressed as mean values ± standard deviation. The differences were tested by the ANOVA test. After adjustments (BMI, age, gender, alcohol intake (g/week), smoking (pack years), Quick index, systolic blood pressure) the statistical significance for TE (p = 0.006) and GGT (p = 0.007) prevailed. Abbreviations are as follows: NAFLD, non-alcoholic fatty liver disease; AF, atrial fibrillation; BMI, body mass index; Quicki (surrogate for insulin resistance, Quicki = 1/[log (fasting insulin)+log (fasting glucose)]); TE, transient elastography; ALT, Alanine aminotransferase; GGT, gamma-glutamyl transpeptidase.

NFS scores in all 129 NAFLD patients at the follow-up visit in year 2013 (mean age 73 years, range 63–83 years; 79 (61%) males; mean BMI 31.7 kg/m^2^, range 20.6–47.4 kg/m^2^) were 0.685 in AF group and 0.129 in non-AF group (before and after adjustments p = 0.038 and p = 0.037, respectively).

The distribution of TE was skewed to the left both visually and according to the Kolmogorov-Smirnov test. The distribution of LAD and NFS were visually and by Kolmogorov-Smirnov test within normal distribution. Thereby, we tested the mean TE values in AF and non-AF subjects with Mann-Whittney U test and TE in the four subgroups (the subjects without NAFLD or AF, with NAFLD but without AF, with both conditions, with AF but without NAFLD) with Kruskal-Wallis non-parametric ANOVA test. However, these did not change the statistical significances (p-values 0.002 and 0.004, respectively, other data not shown).

## Discussion

In the present study, we showed that there is an association between AF and liver stiffness in an elderly Finnish population measured by TE values. The association was observed from two points of view: those with AF had greater TE values than those without, and the AF prevalence and LAD tended to rise with higher TE values. Clinically relevant fibrosis by TE (>8 kPa) was also more prevalent in those with AF than in those without. Moreover, in comparison to non-AF subjects, the higher GGT values in the subjects with AF and higher NFS in NAFLD subjects with AF may also reflect the association. All these results indicate that there is an association between AF and liver stiffness.

According to the earlier studies, NAFLD is an independent risk factor for AF [7, 8]. Furthermore, the cardiovascular risk that NAFLD implies increases by the progression of NAFLD [22]. Thus, the present study is in line with these findings as it shows that the association between NAFLD and AF increases by the progression of liver stiffness.

The mechanisms behind the association between AF and liver stiffness remain unanswered and speculative. The simplest explanation for the association is that these entities share common risk factors and co-morbidities. However, it is interesting that the association remained even after correction for the most important, mutual risk factors (BMI, age, gender, alcohol intake, smoking, Quicki, systolic blood pressure), and there was a ‘biological gradient’, a term known from Hills’ criteria for causality [23], between the prevalence of AF and TE values as well as the LAD and TE values. Thereby, the possible causality can be speculated.

First of all, our earlier report that NAFLD predicts independently AF maybe due to that NAFLD causes diastolic dysfunction, autonomic dysfunction and systemic inflammation, all of which are risk factors for AF [7]. Concurrently, chronic inflammation often leads to liver fibrosis [1, 24–27], characterized by parenchymal necrosis and activation and transformation of hepatic stellate cells and portal fibroblasts into hepatic myofibroblasts. This results in the accumulation of collagen, proteoglycans and glycoproteins and, thereby, changes in the extracellular matrix composition [28, 29]. There is also a solid consensus that AF and inflammation are closely linked [30]. Whether AF is only a consequence of inflammation or a cause as well still remains unconfirmed, but it is plausible that AF may also act as a cause [30–33], and there are also reports that this inflammation is systemic [30, 31, 34]. Taken together, it is possible that AF releases pro-inflammatory factors that take part in the progression of liver stiffness.

At the molecular level, these alterations in liver stiffness are mediated by growth factors, cytokines, chemokines and free radicals. Accumulating evidence has shown that NADPH oxidase (NOX), NOX-4 in particular, has a key role in the production of free radicals, and, thereby, in the pathogenesis of liver fibrosis [28]. Additionally, NOX-4 is also activated in the heart by AF and it contributes to AF-related cardiac fibrosis [35, 36]. Another common factor at the molecular level is galectin-3, which is a multifunctional protein mainly secreted by macrophages. It has an important role in cell apoptosis, proliferation, adhesion, migration and differentiation, as well as in angiogenesis and inflammatory responses. Recent evidence has shown that galectin-3 is activated in fibrotic models and is abnormally increased in fibrotic diseases in general [37] and in the liver in particular [38, 39]. Concurrently, the evidence of the association between galectin-3 and cardiac fibrosis in the heart failure is convincing, [37] but the association of AF and galectin-3 is unclear: according to some reports, galectin-3 is just a bystander in the fibrinogenesis of the heart, with overall co-morbidity, particularly obesity, as the real promoter of fibrosis, whereas some reports point out that even ‘lone AF’, i.e., AF without co-morbidities, increases galectin-3 values [40–43]. Thereby, galectin-3 and NOX-4 may have a role in the fibrinogenesis of both the heart and liver.

AF also produces a hypercoagulable state [44–46]. This is above all a consequence of abnormal blood flow in the left atrium but abnormal changes in blood constituents are also described [45, 46]. There are numerous studies linking hypercoagulable state to liver fibrosis [47–51]. The mechanism is two-way: First, the formation of thrombosis in the liver vasculatures causes ischemia and cell death, which, in turn, leads to inflammation and fibrinogenesis [47, 48]. Second, the hypercoagulability is characterized by the increase of thrombin formation within blood circulation. Fibroblasts and hepatic stellate cells express protease-activated receptors (PAR), which are subdivided into at least four subtypes, PAR-1 to PAR-4. Thrombin and coagulation factor Xa can activate PAR-1 receptors, which leads to stellate cell activation, production of extracellular matrix, tissue remodeling and fibrinogenesis. This phenomenon is called ‘direct stellate cell activation’ [47–51]. Additional evidence of the direct stellate cell activation has come from animal models, in which thrombin caused pro-fibrotic and pro-inflammatory responses in isolated rat atrial fibroblasts [44]. Moreover, PAR-2 activation has also been linked to liver fibrinogenesis [52]. Thus, PAR activation through the AF-produced hypercoagulable state may be one mechanism that links AF to liver fibrosis. It is noteworthy, however, that the use of anticoagulants may attenuate these pro-fibrotic responses [44].

There are some noteworthy limitations to this study.

First, histology is the golden standard for diagnosing liver fibrosis. However, being an invasive procedure, histological samples cannot be taken from the general public without liver morbidities. Thus, we used the liver stiffness surrogates. Of these, NFS is the best externally validated scoring system for NAFLD subjects. However, although only TE is validated to be a proxy measurement of liver stiffness irrespective of its etiology, we observed the association between AF and liver stiffness by TE values as well as by NFS scores. Thereby, we think that the newfound association between AF and liver stiffness is strengthened by the similar findings with different methods.

Second, due to high negative predictive value, TE is mainly used with cut-offs to exclude cirrhotic fibrosis (F4) [21], not as continuous scores as we did. However, according to the Cochrane review, there is a continuum with TE values and fibrosis stage [53].

Third, the TE study subjects selected for the present study were relatively obese, and the reliability of TE has been put into doubt in obesity [18, 21], although some reports see this as a relatively small problem [19] or not a problem at all [9]. However, this was taken into account as subjects with unreliable measurement (5 subjects; 76 were left) due to obesity were excluded from the present study. Moreover, there were quite a few study subjects available for TE analysis, they were senior citizens and mostly men, which may limit the interpretation of the results. Nonetheless, the gender distribution was more even in NFS and, still, the results prevailed.

Fourth, due to the cross-sectional nature of the study, we cannot determine whether the relationship was causal or temporal. However, even after adjustments for mutual risk factors, the association between AF and liver stiffness remained. This may indicate that the association is causal. In addition, there are clinically logical possible pathogenetic mechanisms for the association.

In conclusion, in the present study we introduce the association between atrial fibrillation and liver stiffness, measured either by transient elastography or NAFLD fibrosis score, in an elderly Finnish population. The association is present in subjects with and without NAFLD, which, due to its high prevalence, was viewed separately. To our knowledge, the association between atrial fibrillation and liver stiffness has not been described before. Obviously, further studies are needed to establish these findings and to examine the possible causality.
